# Metabolites and diabetes remission after weight loss

**DOI:** 10.1038/s41387-021-00151-6

**Published:** 2021-02-24

**Authors:** Lydia Coulter Kwee, Olga Ilkayeva, Michael J. Muehlbauer, Nathan Bihlmeyer, Bruce Wolfe, Jonathan Q. Purnell, F. Xavier Pi-Sunyer, Haiying Chen, Judy Bahnson, Christopher B. Newgard, Svati H. Shah, Blandine Laferrère

**Affiliations:** 1 Duke Molecular Physiology Institute, Durham, NC USA; 2 Sarah W. Stedman Nutrition and Metabolism Center, Durham, NC USA; 3 Departments of Surgery and Medicine, Oregon Health & Science University,, Portland, OR USA; 4 New York Obesity Research Center, Division of Endocrinology, Department of Medicine, Columbia University College of Physicians and Surgeons, New York, NY USA; 5 Department of Biostatistics and Data Science, Wake Forest School of Medicine Medical Center, Winston-Salem, NC USA; 6 Department of Pharmacology & Cancer Biology and Division of Endocrinology, Department of Medicine, Duke University, Durham, DC USA; 7 Division of Cardiology, Department of Medicine, Duke University Medical Center, Durham, DC USA

**Keywords:** Obesity, Type 2 diabetes

## Abstract

There is marked heterogeneity in the response to weight loss interventions with regards to weight loss amount and metabolic improvement. We sought to identify biomarkers predictive of type 2 diabetes remission and amount of weight loss in individuals with severe obesity enrolled in the Longitudinal Assessment of Bariatric Surgery (LABS) and the Look AHEAD (Action for Health in Diabetes) studies. Targeted mass spectrometry-based profiling of 135 metabolites was performed in pre-intervention blood samples using a nested design for diabetes remission over five years (*n* = 93 LABS, *n* = 80 Look AHEAD; *n* = 87 remitters), and for extremes of weight loss at five years (*n* = 151 LABS; *n* = 75 with high weight loss). Principal components analysis (PCA) was used for dimensionality reduction, with PCA-derived metabolite factors tested for association with both diabetes remission and weight loss. Metabolic markers were tested for incremental improvement to clinical models, including the DiaRem score. Two metabolite factors were associated with diabetes remission: one primarily composed of branched chain amino acids (BCAA) and tyrosine (odds ratio (95% confidence interval) [OR (95% CI)] = 1.4 [1.0–1.9], *p* = 0.045), and one with betaine and choline (OR [95% CI] = 0.7 [0.5–0.9], *p* = 0.02).These results were not significant after adjustment for multiple tests. Inclusion of these two factors in clinical models yielded modest improvements in model fit and performance: in a constructed clinical model, the C-statistic improved from 0.87 to 0.90 (*p* = 0.02), while the net reclassification index showed improvement in prediction compared to the DiaRem score (NRI = 0.26, *p* = 0.0013). No metabolite factors associated with weight loss at five years. Baseline levels of metabolites in the BCAA and trimethylamine-N-oxide (TMAO)-microbiome-related pathways are independently and incrementally associated with sustained diabetes remission after weight loss interventions in individuals with severe obesity. These metabolites could serve as clinically useful biomarkers to identify individuals who will benefit the most from weight loss interventions.

## Introduction

Obesity is a significant cause of morbidity and mortality. While weight loss strategies are variably effective at a cohort level, there is also marked individual heterogeneity in the amount and sustainability of weight loss^1–3^, as well as in improvements in metabolic and cardiovascular risk factors^4^. There are few known predictors of long-term weight loss responses to lifestyle interventions^5^ or bariatric surgery^6^. Identifying individuals who would benefit the most from a given weight loss strategy would allow for a more personalized approach to weight loss intervention.

Lifestyle interventions generally result in modest weight loss and improvement in glycemic control^7^. In the Action for Health in Diabetes study (Look AHEAD), intensive lifestyle intervention (ILI) resulted in partial or complete type 2 diabetes (T2D) remission in 11.5% of subjects at year 1 and 7.3% at year 4^8^. For individuals with severe obesity, bariatric surgery is the treatment of choice; it results in significant and sustained weight loss and in the amelioration or resolution of most co-morbidities, including T2D^9^. In the Longitudinal Assessment of Bariatric Surgery (LABS), the proportion in diabetes remission at 1, 3, and 5 years were 71.2%, 69.4%, and 64.6% respectively, for Roux-en-Y gastric bypass (RYGB), and 30.7%, 29.3%, and 29.2% for adjustable gastric banding (AGB)^1,10^. However, as with lifestyle interventions, the weight loss and metabolic responses are highly variable among surgery types and individuals^1,9,11^. While several scores to predict T2D remission after RYGB have been validated,^12–14^^,^ and some biomarkers of short-term diabetes remission identified^15–17^, the determinants of metabolic improvement are not fully understood. Moreover, evidence suggests that the beneficial effects of RYGB on T2D may extend beyond calorie restriction and weight loss^18,19^. Metabolomics studies have identified clusters of metabolites that exhibit dramatic changes in response to surgical weight loss, most notably branched chain amino acids (BCAA) and related metabolites^19,20^. The predictive value of other biomarkers, such as bile acids^21,22^ or others linked to the gut microbiome^23^, remains uncertain.

Here, we wished to capitalize on important clinical trials of surgical (LABS) and lifestyle (Look AHEAD) weight loss interventions to investigate metabolic pathways underlying clinical outcomes in individuals with obesity and T2D. We sought to better understand the heterogeneity of metabolic responses between individuals and identify baseline metabolic biomarker predictors of sustained T2D remission and weight loss. Based on our previous work, we hypothesized that pre-intervention circulating plasma levels of BCAA and related metabolites, and metabolites influenced by the microbiome (i.e. choline metabolites, bile acids, and short-chain fatty acids (SCFA)), would be associated with T2D remission and weight loss amount. We further hypothesized that individual metabolites or metabolite clusters would provide incremental predictive capability for T2D remission, above and beyond currently known clinical predictors. To achieve these goals we applied targeted, quantitative mass spectrometry-based metabolomic profiling to samples collected prior to intervention from a subgroup of well-characterized participants from the LABS^9^ and Look AHEAD^24^ studies with follow up at 2 and 4 to 5 years after intervention.

## Methods

### Study populations

#### The LABS Study

is a multicenter observational cohort study of individuals with obesity undergoing first-time bariatric surgery that was conducted at 10 hospitals throughout the U.S. A total of 2458 participants were studied from 2006 and 2009 until January 31, 2015. In-person research assessments at pre-surgery, 2 and 5 years included body weight, fasting blood sampling, and assessment of comorbidities, and results were previously reported^1,9,10^. All participants signed a consent form approved by their center’s Institutional Review Board (IRB).

#### Look AHEAD

was a multi-center, randomized controlled trial designed to test the effects on cardiovascular morbidity and mortality of an ILI intended to produce 5–10% weight loss. A total of 5145 men and women with T2D were enrolled and randomly assigned, with equal probability, to ILI versus diabetes support and education (DSE) in 16 centers throughout the U.S. Measured height, weight, and fasting plasma glucose and HbA1c were assessed at baseline (August 2001–April 2004) and yearly thereafter, with the year 4 visit occurring between August 2005 and April 2008. All participants signed a consent form approved by their center’s IRB. The beneficial effects of the intervention on diabetes remission and amelioration of other co-morbidities have been reported^8,25–28^.

### Sample selection

Pre-intervention samples from each study were selected based on two distinct post-intervention phenotypes: (1) diabetes remission status at both 2 and 5 years (LABS) or at both 2 and 4 years (Look AHEAD) (*n* = 173 total, with 83% power to detect a pre-specified effect size of 0.4); and (2) extremes of amount of weight loss at 5 years (*n* = 151 from LABS cohort only, with 79% power to detect a pre-specified effect size of 0.4). All samples were stored in NIH repositories following strict study protocols.

#### Diabetes remission phenotype

All subjects from LABS and LookAHEAD included in the diabetes remission phenotype analysis had diabetes at baseline, defined as HbA1c ≥ 6.5% and/or the use of diabetes medications. After the interventions, diabetes status was defined at each study visit as persistent diabetes (HbA1c ≥ 6.5% and/or the use of diabetes medications), partial remission (HbA1c from 5.7% to 6.4% and not on diabetes medications), or complete remission (HbA1c < 5.7% and not on diabetes medications).

Criteria for LABS participants included in this study were: (1) available samples and clinical data on diabetes status at all timepoints; (2) no revision surgery after initial surgery; (3) no pregnancy through year 5. Diabetes duration was not an inclusion criterion. Remitters were defined as those with complete remission at both the 2 year and 5 year visits. Non-remitters were defined as those with persistent diabetes at both the 2 year and 5 year visits. Of LABS subjects meeting these definitions (*n* = 126), we selected 47 remitters and 46 non-remitters for this study, based on greatest sample availability (Supplementary Figure).

Inclusion criteria for Look AHEAD participants in this study were: (1) available samples and clinical data at all timepoints; (2) baseline BMI ≥ 35 kg/m^2^ (to mirror the bariatric BMI threshold); (3) no bariatric surgery through year 4; (4) known diabetes duration ≤ 5 years. Remitters were defined as individuals with complete or partial remission at both 2 and 4 years; non-remitters were defined as those with persistent diabetes at both 2 and 4 years. Look AHEAD subjects meeting these definitions were matched in remitter/non-remitter pairs based on intervention type (ILI/DSE), pre-intervention insulin use, HbA1c (within 0.5%), and diabetes duration (within 2 years). Of 50 identified matched pairs, we randomly selected 40 pairs of diabetes remitters/non-remitters for this study (Supplementary Figure).

#### Extremes of weight loss phenotype

LABS participants were selected based on the percentage of weight lost at five years post-surgery. Specifically, individuals in the top or bottom 13^th^ percentile of weight loss within each intervention group (RYGB and AGB) were selected to represent best or worst weight loss outcomes, respectively. This sample selection approach yielded the desired sample size while proportionally including samples from both intervention groups in the outcomes: 55 RYGB subjects with “best” weight loss at 5 years (40.6–54.4% weight loss) and 20 AGB (30.4–48.1% weight loss), along with 56 RYGB subjects with worst weight loss (loss of 17.2%–gain of 7.0%) and 20 AGB (loss of 1.9%–gain of 18.9%), for a total of 151 individuals.

### Metabolomic profiling and traditional laboratory measures

A total of 135 metabolites were quantitatively measured in frozen, fasting plasma from baseline (pre-intervention) samples using mass-spectrometry-based methods. This included trimethylamine-N-oxide (TMAO), choline, betaine, 2-aminoadipic acid, β-amino isobutyric acid, 3-hydroxyisobutyric acid, branched chain ketoacids (BCKA), 13 bile acids, 15 amino acids, 45 acylcarnitines, 21 ceramides and 29 sphingomyelins. Additionally, traditional laboratory methods were used to measure non-esterified fatty acids (NEFA), total ketones, 3-hydroxybutyric acid (3-HB), lactate, high-sensitivity C-reactive protein (hs-CRP), glucose, insulin and HbA1c For detailed methodology, including calculation of HOMA-IR (homeostatic model assessment of insulin resistance) and HOMA-B (homeostatic model assessment of β-cell function), please see Supplementary Information.

### Statistical analysis

#### Metabolomics

Our analysis strategy included both pre-specified hypotheses based on prior work in obesity and diabetes^20,29–31^, and a full unbiased discovery approach^32^. For the pre-specified analyses, we considered the following metabolites and metabolite summary measures: BCAA (valine, leucine/isoleucine), BCKA (ketoisocaproate [KIC], ketomethylvalerate [KMV], ketoisovalerate [KIV]), the BCKA/BCAA ratio, and 2-aminoadipic acid (2-AAA). For discovery analyses, we used principal components analysis (PCA) with varimax rotation to create standardized, normally-distributed summary factors for analysis, as we have done previously^33–35^. This approach results in dimensionality reduction given collinearity of many of the metabolites and allows the identification of metabolic pathways represented by metabolites clustering in a factor. Metabolites with >25% of values below the lower limits of quantification were excluded (*n* = 5 acylcarnitines). Ceramide/sphingomyelin profiling was performed on a subset of 125 samples identified as remitters or non-remitters, with priority given to LABS participants (*n* = 91 included) and 17 Look AHEAD pairs randomly selected (*n* = 34). Factor construction was performed on the ceramide/sphingomyelin panel separately from all other metabolites, due to the smaller number of samples (*n* = 125 vs. *n* = 301 for other metabolites). We retained factors for use in downstream analysis based on the Kaiser criterion (eigenvalue > 1). Each factor was annotated based on known biological pathways or metabolite classes represented by the metabolites heavily loaded on the factor (absolute value of factor loading >0.4). For interpretability, factor signs were set so that the majority of heavily loaded metabolites had positive loadings on a factor.

#### Analyses for diabetes remission phenotype

We tested the pre-specified baseline metabolites and PCA-derived factors for association with remission using logistic regression in the 173 remitters and non-remitters from LABS and Look AHEAD. To identify important clinical/laboratory covariates, we used univariate logistic regression to test for association between remission status and the following variables: age, race, sex, total cholesterol, HDL (high-density lipoproteins), LDL (low-density lipoproteins), triglycerides, HbA1c, glucose, insulin, HOMA-IR, HOMA-B, RYGB (vs. all other interventions), weight, insulin use, metformin use, the number of non-insulin diabetes medications, and percent weight change at 2 years. Stratified sensitivity analyses were conducted in the following subgroups: (1) LABS subjects (*n* = 93); (2) Look AHEAD subjects (*n* = 80); (3) RYGB subjects (*n* = 71); (4) non-RYGB subjects (*n* = 102: 22 LABS, 80 Look AHEAD).

To assess the incremental value of metabolomic data to predict diabetes remission on top of a clinical model, model fit (C-statistic)^36^ and net reclassification index (NRI)^37–39^ analyses were used, comparing a model only incorporating the clinical and laboratory values significant in univariate analysis to a model inclusive of clinical variables and metabolomic predictors significant in univariate analyses (*p* < 0.05). For NRI, subjects’ risk was binned into tertiles (low: 0–0.33; medium: 0.34–0.66; high: 0.67–1). We also examined the incremental value of metabolomic data in relation to the clinical DiaRem score predicting remission after RYGB^12^. We estimated the probability of remission using a logistic regression model containing either the DiaRem score only, or the DiaRem score and metabolomic predictors significant in univariate analyses. After binning the scores as done previously^12^, we established cutoffs for the NRI based on predicted probabilities of remission in our sample (bin 1: 0.64–1; bin 2: 0.38–0.63; bin 3: 0.18–0.37; bin 4: 0.09–0.17; bin 5: 0–0.08).

Finally, we used linear regression to test for associations between baseline values of metabolites significantly associated with remission status and change in diabetes-related clinical variables (HbA1c, HOMA-B, and HOMA-IR) from pre-intervention to two years post-intervention, adjusting for baseline levels of the clinical variable in the model.

#### Analyses for extremes of weight loss phenotype

We conducted bivariate tests of association between baseline metabolites/factors and weight loss status (highest vs. lowest percentage weight lost at 5 years). For these analyses, we used logistic regression to test the pre-specified metabolites and non-ceramide/sphingomyelin factors in 151 LABS subjects.

Model assumptions were met for logistic and linear regression. All analyses were performed using R version 4.0.1 (https://www.R-project.org/).

## Results

Baseline clinical and demographic characteristics of selected individuals are shown in Tables 1 and 2. Participants were predominantly female and white. Remitters had lower HbA1c and glucose levels compared with non-remitters, but otherwise did not differ in age, BMI, waist circumference, lipid levels, and clinical metabolite factors at baseline (Table 1). Remitters used fewer diabetes medications at baseline and exhibited greater weight loss over time compared to non-remitters. As a consequence of a stricter definition of diabetes remission in the LABS study, HbA1c and fasting glucose at baseline, 2, and 4/5 years differed between the Look AHEAD and LABS remitter’s cohorts. However, the change in both glucose and HbA1c from baseline to 2 years and 4 or 5 years was not significantly different between LABS and Look AHEAD remitters. LABS study participants at the extremes of weight loss at five years had similar baseline characteristics (Table 2).

**Table 1:** Baseline demographics and clinical characteristics of individuals with and without type 2 diabetes remission, from LABS and LookAHEAD cohorts combined.

	Non-Remitters	Remitters	*p*
*n*	86	87	
Intervention (%)			0.03
AGB	17 (19.8)	5 (5.7)	
RYGB	29 (33.7)	42 (48.3)	
DSE	7 (8.1)	7 (8.0)	
ILI	33 (38.4)	33 (37.9)	
Age (years)	54.95 (8.75)	53.28 (9.25)	0.22
Female (%)	62 (72.1)	56 (64.4)	0.35
White (%)	62 (72.1)	73 (83.9)	0.09
Baseline HbA1c (%)	7.21 (1.27)	6.44 (0.79)	<0.001
HbA1c at yr 2 (%)	6.73 (1.02)	5.38 (0.40)	<0.001
HbA1c at yr 4/5 (%)	7.12 (1.27)	5.46 (0.43)	<0.001
Baseline BMI (kg/m2)	43.5 (6.6)	44.6 (7.6)	0.33
Weight change at yr 2 (%)	−14.5 (13.4)	−23.8 (13.1)	<0.001
Weight change at yr 4/5 (%)	−12.6 (14.5)	−20.8 (13.3)	<0.001
Waist circumference (cm)	128.4 (16.4)	130.9 (17.2)	0.32
Fasting glucose (mg/dl)	144.4 (51.0)	127.7 (31.5)	0.01
Fasting insulin (uU/ml)	25.1 (22.4)	24.4 (14.4)	0.80
HOMA-IR	7.54 (8.26)	7.68 (4.61)	0.9
HOMA-B	113.9 (75.0)	165.8 (138.1)	0.008
CHOL (mg/dl)	183.4 (36.9)	184.9 (38.1)	0.78
HDL (mg/dl)	42.9 (10.1)	41.4 (11.3)	0.35
LDL (mg/dl)	104.4 (34.4)	102.8 (31.8)	0.76
TRIG (mg/dl)	188.4 (103.4)	222.9 (173.0)	0.11
Serum creatinine (mg/L)	0.79 (0.25)	0.82 (0.27)	0.47
hs-CRP (mg/L)	0.75 (1.03)	0.74 (0.83)	0.92
Metformin use at baseline (%)	55 (64.7)	48 (55.2)	
	0.26		
# Non-insulin T2D meds at baseline (%)			0.03
0	13 (15.3)	24 (27.6)	
1	40 (47.1)	42 (48.3)	
2	22 (25.9)	19 (21.8)	
3	10 (11.8)	2 (2.3)	
Using insulin at baseline (%)	24 (27.9)	4 (4.6)	<0.001

Data presented as mean (SD) for continuous variables and *n* (%) for categorical variables. *AGB* adjustable gastric banding, *RYGB* Roux-en-Y gastric bypass, *ILI* intensive life style intervention, *DSE* diabetes education.

**Table 2:** Baseline demographics and clinical characteristics of individuals in best and worst weight loss groups (LABS study only).

	Best weight loss	Worst weight loss	*p*
*n*	75	76	
Intervention (%)			
AGB	55 (73.3)	56 (73.7)	
RYGB	20 (26.7)	20 (26.3)	
Age (years)	46.0 (11.4)	47.7 (10.3)	0.34
Female (%)	63 (84.0)	60 (78.9)	0.56
White (%)	63 (84.0)	70 (92.1)	0.20
Baseline HbA1c (%)	5.88 (0.90)	5.96 (1.10)	0.63
HbA1c at yr 2 (%)	5.14 (0.41)	5.46 (0.75)	0.001
HbA1c at yr 5 (%)	5.14 (0.45)	5.80 (1.06)	<0.001
Baseline BMI (kg/m2)	49.2 (7.8)	47.3 (6.9)	0.11
Weight change at yr 2 (%)	−40.0 (11.0)	−18.6 (11.6)	<0.001
Weight change at yr 5 (%)	−42.1 (5.9)	−8.5 (8.7)	<0.001
Waist circumference (cm)	134.5 (16.9)	132.8 (16.8)	0.54
Fasting glucose (mg/dl)	106.8 (29.3)	110.2 (35.6)	0.53
Fasting insulin (uU/ml)	26.5 (25.7)	21.5 (14.2)	0.14
HOMA-IR	6.38 (4.47)	5.79 (4.68)	0.44
HOMA-B	227.4 (160.0)	212.0 (137.1)	0.54
CHOL (mg/dl)	184.1 (32.1)	182.9 (34.3)	0.82
HDL (mg/dl)	45.3 (11.2)	43.6 (11.8)	0.38
LDL (mg/dl)	108.7 (27.1)	109.7 (28.6)	0.83
TRIG (mg/dl)	150.5 (63.3)	147.8 (68.7)	0.80
Serum creatinine (mg/L)	0.78 (0.28)	0.75 (0.22)	0.44
hs-CRP (mg/L)	1.26 (1.42)	0.94 (1.13)	0.13
Metformin use at baseline (%)	11 (17.2)	20 (29.9)	0.13
# Non-insulin diabetes meds at baseline (%)			0.35
0	45 (77.6)	39 (62.9)	
1	7 (12.1)	14 (22.6)	
2	5 (8.6)	7 (11.3)	
3	1 (1.7)	2 (3.2)	
Using insulin at baseline (%)	5 (6.9)	4 (5.5)	0.98

Data presented as mean (SD) for continuous variables and *n* (%) for categorical variables. *AGB* adjustable gastric banding, *RYGB* Roux-en-Y gastric bypass.

### Clinical variables and baseline metabolites associated with diabetes remission

PCA identified 29 factors for analysis: eight factors were derived from the 50 ceramide/sphingomyelin species (referred to as CS factors 1–8, based on *n* = 125 samples), and 21 factors were derived from the remaining 81 metabolites (referred to as factors 1–21, based on n301 samples). These factors, and the primary metabolites loaded on each, are described in Supplemental Table 1.

**Fig. 1:**
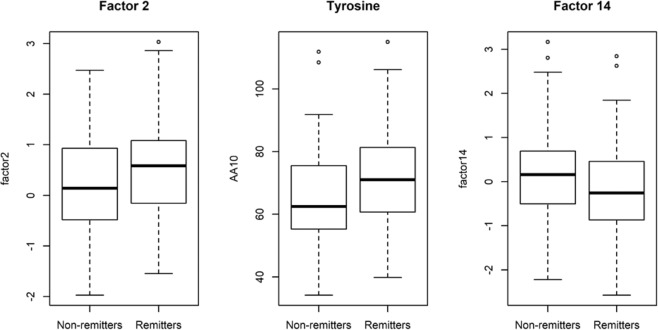
Pre-intervention levels of significant factors. Factor 2 (BCAA, tyrosine, phenylalanine, methionine, arginine and histidine), factors 14 (betaine and choline) and tyrosine in individual with (remitters) or without (non-remitters) remission of type 2 diabetes after weight loss interventions.

Results of univariate analyses of diabetes remission are given in Table 3, including all tested clinical variables and any significant pre-specified individual metabolites or metabolite factors (*p* < 0.05). Consistent with prior reports^8,11,40,41^, baseline HbA1c, glucose, HOMA-B, use of insulin, number of non-insulin diabetes medications, and percent weight change at two years were all associated with remission in the combined LABS and Look AHEAD cohorts. Baseline levels of two metabolite factors were nominally associated with remission: factor 2 (primarily composed of BCAA and the aromatic amino acids phenylalanine and tyrosine, which share a large neutral amino acid transporter with BCAA, along with methionine, arginine, and histidine) (odds ratio (95% confidence interval) [OR (95% CI)] = 1.37 [1.01–1.88], *p* = 0.045, Fig. 1); and factor 14 (primarily composed of betaine and choline, which are metabolized by gut bacteria to yield trimethylamine (TMA), which in turn is converted to TMAO by the liver (OR [95% CI] = 0.69 [0.50–0.94], *p* = 0.02, Fig. 1). No pre-specified metabolites or metabolite factors were significantly associated with remission after adjusting for multiple comparisons by controlling the false discovery rate (FDR) at 5%.^42^

**Table 3:** Association of clinical features and selected metabolite factors with diabetes remission at 2 and 4/5 years.

	OR (95% CI)	*p*	FDR-adjusted *p*
Age (years)	0.98 (0.95–1.01)	0.22	0.63
Ancestry (white vs. all other)	2.02 (0.97–4.32)	0.063	0.33
Female	0.7 (0.36–1.33)	0.28	0.63
Total cholesterol^a^	1.04 (0.77–1.41)	0.78	0.93
HDL^a^	0.87 (0.63–1.17)	0.35	0.63
LDL^a^	0.95 (0.69–1.3)	0.75	0.93
Triglycerides^a^	1.3 (0.95–1.85)	0.12	0.50
HbA1c (%)	0.43 (0.28–0.62)	2.8 ×10^−5^	0.00081
Glucose^a^	0.64 (0.44–0.9)	0.015	0.15
Insulin^a^	0.96 (0.7–1.31)	0.8	0.93
HOMA-IR	1.0 (0.95–1.06)	0.9	0.97
HOMA-B	1.01 (1.0–1.01)	0.013	0.15
RYGB (vs. all other)	1.83 (1.0–3.41)	0.053	0.33
Weight at baseline (kg)	1.0 (1.0–1.01)	0.22	0.63
% Weight change at 2 years	0.95 (0.93–0.97)	2.4 ×10^−5^	0.00081
Taking insulin at baseline	0.12 (0.04–0.34)	0.00023	0.0044
Taking metformin at baseline	0.67 (0.36–1.24)	0.2	0.63
# Non-insulin T2D meds at baseline	0.59 (0.4–0.86)	0.007	0.10
Factor 2 (BCAA/aromatic AA)	1.37 (1.01–1.88)	0.045	0.33
Factor 14 (betaine/choline)	0.69 (0.50–0.94)	0.02	0.17

^a^Odds ratios are for 1 standard deviation of the indicated clinical feature or metabolite.

Results are shown from all tested clinical characteristics, along with nominally significant metabolites/factors (*p* < 0.05). An odds ratio (OR) > 1 indicates that higher levels of the tested variable is associated with increased odds of diabetes remission; OR < 1 indicates that higher levels are associated with decreased odds of remission. Weight loss at 2 years is negative and therefore is associated with an increased odds of remission.

*HDL* high-density lipoproteins, *LDL* low-density lipoproteins, *HbA1c* hemoglobin A1c, *HOMA-IR* homeostatic model assessment of insulin resistance, *HOMA-B* homeostatic model assessment of β-cell function, *RYGB* Roux-en-Y gastric bypass, *T2D* type 2 diabetes, *BCAA* branched-chain amino acids, *AA* amino acids.

To determine which individual metabolites might be driving these factor associations, we tested each metabolite heavily loaded in factors 2 and 14 individually for association with remission. In factor 2, only tyrosine levels were associated with remission status, with higher levels in remitters than non-remitters (*p* = 0.0035, Fig. 1). In factor 14, levels of both betaine and choline were slightly lower in remitters than non-remitters, but the difference was not statistically significant for either metabolite (*p* = 0.27 and *p* = 0.07, respectively). Of note, none of the pre-specified metabolites from our hypothesis-based approach (BCAA, BCKA, 2-AAA) were individually associated with diabetes remission (Supplementary Table 2).

In subgroup analyses stratified by study and by intervention (RYGB vs. all others), factor 2 demonstrated consistent effects across all subgroups (OR = 1.37–1.58, *p* = 0.03–0.18, Supplementary Table 3). In contrast, factor 14 was significantly associated with remission status only in the Look AHEAD and non-RYGB subgroup analyses (OR = 0.38, *p* = 0.0014 and OR = 0.53, *p* = 0.0064, respectively) and was not significant in the LABS (RYGB + AGB) and RYGB-only subgroups (OR = 0.97, *p* = 0.89 and OR = 0.94, *p* = 0.79, respectively). A ceramide/sphingomyelin factor (CS factor 4, composed primarily of glucosylceramides and ceramides), not significant in the full combined analysis, was nominally significant in the LABS and RYGB subgroup analyses: (OR = 1.58, *p* = 0.045 and OR = 1.92, *p* = 0.026, respectively). Full results from the subgroup analyses for these metabolite factors, along with all clinical variables, are given in Supplementary Table 3.

### Incremental predictive capabilities of metabolites over a clinical model for diabetes remission

Clinical covariates associated with remission in univariate analyses were then included in a full clinical model, with the exception of glucose (highly correlated with HbA1c) and HOMA-B. The clinical model therefore included pre-intervention HbA1c, use of insulin, number of non-insulin diabetes medications and percent weight loss at 2 years. When significant metabolite factors were included in this clinical model, factor 14 remained associated with remission (*p* = 0.0037) while the significance of the association with factor 2 was attenuated somewhat (*p* = 0.051) (Table 4). The odds ratios for both factors became greater in the multivariable models compared to the univariate models, suggesting that the association between these metabolite factors and remission status is independent of these clinical variables. The results were similar when both factors were included in the same multivariable model, suggesting that they are reporting on different biological associations (OR [95% CI] = 1.58 [1.02–2.52], *p* = 0.046 and 0.51 [0.32–0.79], *p* = 0.0034 for factors 2 and 14, respectively).

**Table 4:** Multivariable models with clinical features and significant metabolite factors.

	Clinical model + factor 2		Clinical model + factor 14		Clinical model + factors 2 and 14		DiaRem + factor 2		DiaRem + factor 14		DiaRem + factors 2 and 14	
	OR (95% CI)	*p*	OR (95% CI)	*p*	OR (95% CI)	*p*	OR (95% CI)	*p*	OR (95% CI)	*p*	OR (95% CI)	*p*
**HbA1c**	0.40 (0.23–0.66)	0.0008	0.39 (0.22–0.64)	0.00043	0.35 (0.19–0.59)	0.00029						
**Insulin use**	0.06 (0.01–0.25)	0.0002	0.05 (0.01–0.18)	3.8×10^−5^	0.07 (0.01–0.26)	0.00032						
**# Non-insulin T2D drugs**	0.41 (0.24–0.67)	0.0007	0.39 (0.22–0.64)	0.00037	0.37 (0.21–0.61)	0.00026						
**% Weight change at 2** **y**	0.91 (0.87–0.94)	7.5×10^−8^	0.91 (0.88–0.94)	2.1×10^−7^	0.91 (0.87–0.94)	1.3×10^−7^						
**DiaRem score**							0.81 (0.73–0.89)	4.4×10^−5^	0.79 (0.7–0.87)	8.1×10^−6^	0.79 (0.71–0.88)	2.1×10^−5^
**Factor 2**	1.52 (1–2.35)	0.051			1.58 (1.02–2.52)	0.046	1.17 (0.83–1.67)	0.37			1.15 (0.8–1.68)	0.44
**Factor 14**			0.52 (0.33–0.8)	0.0037	0.51 (0.32–0.79)	0.0034			0.60 (0.42–0.84)	0.0036	0.60 (0.42–0.84)	0.004

An odds ratio (OR) > 1 indicates that higher levels of the clinical variable or metabolite factor is associated with increased odds of diabetes remission; OR < 1 indicates that higher levels are associated with decreased odds of remission. Weight loss at 2 years is negative and therefore is associated with an increased odds of remission. *HbA1c* hemoglobin A1c, *T2D* type 2 diabetes.

**Fig. 2:**
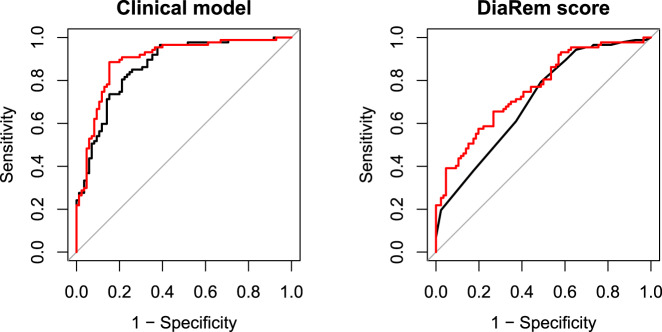
Receiver operating characteristic (ROC) curves for clinical models alone (black) or with significant metabolite factors (red). Factor 14 (betaine and choline) and factor 2 (BCAA, tyrosine, phenylalanine, methionine, arginine and histidine). On the left, the black line shows the ROC curve for a clinical model including baseline HbA1c level, insulin use, number of non-insulin type 2 diabetes medications, and weight change 2 years post-intervention. On the right, the black line shows the ROC curve for the DiaRem score.

We then tested the incremental predictive capabilities of these metabolite factors over both our constructed clinical model of diabetes remission and over the DiaRem score using the C-statistic and net reclassification index (NRI). For the constructed clinical model alone, the C-statistic was 0.87; addition of individual metabolite factors did not significantly improve prediction (C-statistic=0.88 for each), but addition of both factors resulted in a modest improvement in the C-statistic from 0.87 to 0.90 (Fig. 2, *p* = 0.022). In NRI analyses, adding both factors into the constructed clinical model led to a small, statistically non-significant NRI (0.12, *p* = 0.06). In contrast, we did not see a statistically significant improvement in the C-statistic when adding factors 2 and 14 to a model using the DiaRem score: including both factors led to an increase from 0.71 to 0.76 (Fig. 2, *p* = 0.08). However, the NRI showed a significant benefit to including both metabolite factors in a model containing the DiaRem score (NRI = 0.26, *p* = 0.0013), most of which came from improving prediction for non-remitters (NRI- = 0.24).

In addition to remission status, we also assessed association between baseline levels of these two factors (2 and 14) with change in HbA1c, HOMA-B and HOMA-IR over two years. Only pre-intervention levels of factor 14 were associated with change in HbA1c (*p* = 0.010) at 2 years, with lower baseline levels of pre-intervention betaine and choline associated with decreases in HbA1c at 2 years. Neither factor was associated with change in HOMA-B or HOMA-IR.

### Association of baseline metabolites with extremes of weight loss at 5 years

Of all the pre-intervention candidate metabolites and metabolite factors tested, none were associated with extremes of weight loss at 5 years (Supplementary Table 4).

## Discussion

Using targeted metabolomic profiling of 135 metabolites, we have identified discrete metabolomic signatures measured at pre-intervention that are associated with subsequent sustained diabetes remission at 2 and 4 to 5 years after surgical or lifestyle weight loss interventions in individuals with severe obesity. Importantly, these metabolites are associated with diabetes remission independent of, and incremental to, clinical factors that predict remission, including the DiaRem score. However, we found no specific signature associated with extremes of weight loss at 5 years in LABS.

Specifically, clusters of metabolites comprised of BCAA, tyrosine and other amino acids (factor 2) and of betaine and choline (factor 14) were nominally associated with diabetes remission, but among the metabolites in factor 2, only tyrosine showed association with remission as an individual metabolite. Our group has previously identified a PCA factor containing BCAA, phenylalanine and tyrosine that is strongly associated with insulin resistance^20^ and subsequent work identified possible mechanistic links between BCAA, their metabolism, and metabolic disease pathogenesis^43^. Two other studies, one using samples from the Framingham Heart Study, also identified BCAA, phenylalanine and tyrosine as predictors of incident diabetes. Similar to the results reported here, those studies found that tyrosine was the baseline metabolite most strongly associated with an increased risk of diabetes^44,45^. Moreover, we and others have shown that significant changes in concentrations of BCAA and related metabolites occur in association with improved insulin sensitivity after surgical weight loss^19,46–48^.

Interestingly, individual BCAA and BCKA did not associate with the diabetes remission phenotype, suggesting that despite the fact that these metabolites are associated cross-sectionally with obesity, insulin resistance and diabetes, and have been shown to predict change in insulin resistance with behavioral and bariatric weight loss interventions, they are less useful for predicting the clinical phenotype of diabetes remission. We also note that baseline circulating bile acids and SCFA, both known to change after RYGB^22,49^, were not predictive of the diabetes remission phenotype, contrary to a previous short-term study in 38 Chinese patients^15^. Given the relatively small sample size of our study and use of a binary trait of remission, our power may have been limited to detect these associations.

Factor 14 is composed of betaine and choline, both of which are precursors for gut microbial synthesis of TMA, which is rapidly oxidized to TMAO in the liver^50^. TMAO enhances the accumulation of cholesterol in macrophages and foam cells in artery walls; TMAO circulating concentrations, like choline and betaine, are associated with an increased risk of adverse cardiovascular events, after adjusting for traditional risk factors^51,52^. Choline can also be oxidized to betaine in the liver and the kidneys^53^. Betaine is involved in methylation reactions and detoxification of homocysteine^54^. TMAO has also been associated with diabetes risk^55^, and plasma choline levels have been associated with glucose levels^56^, although none of these choline-derived biomarkers have been associated with incident diabetes phenotypes. Interestingly, TMAO did not cluster with choline and betaine in a PCA-derived factor, and TMAO alone did not associate with diabetes remission. The lack of association between TMAO and remission could be because betaine and choline metabolites are more functionally relevant to the clinical state of diabetes, supported by a study that showed that plasma choline changed to a greater extent than TMAO during an oral glucose tolerance test^56^, because the metabolism of choline and betaine occurs in multiple organs, while formation of TMAO occurs in the liver^57^, or because of the greater intra-individual variation of circulating TMAO compared to betaine and choline^58^. Regardless, our results suggest that choline and betaine particularly could play a role in diabetes remission in individuals with obesity, particularly after non-RYGB weight loss. Although this may seem contradictory given known effects on the microbiome in response to RYGB^49^, perhaps this effectively reflects a washing out of inter-individual differences in metabolic risk related to the gut microbiome in patients who have RYGB.

CS factor 4, composed primarily of a variety of glucosylceramides, showed an association with diabetes remission after RYGB. Although this association was only seen in subgroup analyses and should be treated cautiously, it also recapitulates previously reported associations between sphingomyelin and diabetes in humans^59^ and glucosylceramide levels and insulin sensitivity in both humans and animal models^60^.

The strengths of our study include the utilization of two national, multi-center studies with two modes of weight loss interventions that provided detailed longitudinal phenotyping of key clinical variables. Of the few studies using metabolomics to predict outcome, most are small scale (10–40 subjects) and short term (1–12 months)^15–17^. Our targeted metabolomics assays, by virtue of inclusion of internal standards, are both accurate and quantitative, as opposed to non-targeted platforms which are comprehensive but lack absolute quantification. Finally, the study focused on sustained diabetes remission, a highly relevant clinical phenotype, and identified metabolite factors that (1) recapitulate known biological associations with glucose regulation and (2) show evidence for incremental improvement over both our constructed clinical model and the DiaRem score. We are not aware of other studies that have shown BCAA or gut-related metabolites improving upon existing clinical models for long-term diabetes remission.

Several limitations, however, should be noted. While we drew from two important clinical weight loss intervention trials, our sample size was limited due to availability of participants with appropriate biospecimens, stringency of our criteria for sustained remission status, and budget limitations. Additionally, the associations we present here between metabolite factors and remission are nominally significant but do not survive FDR adjustment for multiple tests. However, we decided a priori to report nominally significant associations as our study had two goals: (1) to examine associations with BCAA-related metabolites, building on the backbone of existing strong biological and epidemiological data in the literature about the role of BCAA pathway metabolites, and (2) to explore possible associations in an unbiased fashion across a range of other metabolites. We believe the nominal associations reported here are biologically relevant, extend existing understanding of the role of BCAAs, and may suggest plausible mechanisms of action for gut-related metabolites. The lack of information about diet, vitamin supplements and antibiotics, all modulators of the microbiome and the metabolome, along with the lack of an additional validation cohort in which to replicate these findings, is also a limitation. Diabetes duration, an important predictor, was not available for LABS participants. Also, whereas our results suggest that factors 2 and 14 may be incrementally associated with diabetes remission, the absolute change in model fit and risk reclassification is modest when the factors are added either to our constructed clinical model or to the established DiaRem score.

In conclusion, using two large national weight loss multi-center studies in individuals with severe obesity, we have identified circulating baseline biomarkers associated with diabetes remission with independent association as well as incremental predictive capabilities when added to a clinical model.

## Supplementary information

Supplemental material
