# Supplementary material for: Metabolites and diabetes remission after weight loss
Source: Nutr Diabetes. 2021 Feb 24;11:10. doi: 10.1038/s41387-021-00151-6 (PMC7904757; doi:10.1038/s41387-021-00151-6)
Supplement: Supplementary file 1 — Supplemental material [file 41387_2021_151_MOESM1_ESM.docx]

**Supplementary Information**

Abbreviations

Methods

- Study population

- Traditional laboratory measures

- Metabolomic profiling

Supplementary Figure: Consort diagram of sample selection

Supplementary Table 1: PCA-derived factors, and the primary metabolites in each

Supplementary Table 2: Association of candidate metabolites with diabetes remission status

Supplementary Table 3: Association of candidate metabolites with extremes of weight loss

References

**Abbreviations**

AGB: Adjustable gastric banding

BCAA: branched-chain amino acids (valine, leucine/isoleucine)

BCKA: branched-chain ketoacids (alpha-ketoisocaproic (KIC), alpha-ketoisovaleric (KIV), and

alpha-keto-beta-methylvaleric (KMV) acids)

2-AAA: 2-amino adipic acid

DSE: diabetes education

HbA1c: hemoglobin A1c

HDL: high-density lipoproteins

HOMA-IR: homeostatic model assessment of insulin resistance

HOMA-B: homeostatic model assessment of β-cell function

LSI: lifestyle intervention

LDL: low-density lipoproteins

RYGB: Roux-en-Y gastric bypass surgery

T2D: type 2 diabetes

**Methods**

Study populations

***LABS.*** The Longitudinal Assessment of Bariatric Surgery (LABS) Consortium is a multicenter observational cohort study of obese individuals undergoing bariatric surgery that was conducted at 10 U.S. hospitals in six geographically diverse clinical centers. Adults undergoing first-time bariatric surgical procedures as part of routine clinical care were recruited between 2006 and 2009 and followed up through January 31, 2015. Research assessments were conducted within 30 days prior to the surgical procedure and at approximately 6 months, 12 months, and annually following the surgical procedure up to 7 years through the study end date. Research assessments at pre-surgery, 2 and 5 years were conducted in person. Sociodemographic characteristics and race/ethnicity were self-reported. During in-person assessments, weight was measured on a standard scale (Tanita Body Composition Analyzer, model TBF-310). Comorbidities were-assessed as previously described ^1^. Women reporting polycystic ovarian syndrome who did not meet laboratory criteria for diabetes and who were not receiving a diabetes medication other than metformin were considered to not have diabetes. The Institutional Review Boards at each center approved the protocol, and all participants gave written informed consent to participate in the study. Beneficial results from surgical weight loss in LABS participants, including effect on diabetes remission, have previously been reported ^2-5^.

***LookAHEAD***. A total of 5,145 men and women with T2D were enrolled in the Look AHEAD randomized clinical trial of lifestyle intervention at 16 centers throughout the U.S. ^6, 7^. Eligible individuals for the parent Look AHEAD trial had T2D, were 45–76 years of age, and had a body mass index (BMI) ≥25 kg/m2. Additional eligibility criteria have been reported and included applicants’ completing a graded exercise test (to ensure they would be able to participate in a lifestyle intervention that included exercise) and a test of behavioral adherence ^8^. Those who remained eligible were randomly assigned, with equal probability, to intensive lifestyle intervention (ILI) or diabetes support and education (DSE), as described previously ^6, 7^ . Participants in both groups received all usual medical care from their own primary care providers.

Self-reported behavioral and demographic risk factors along with measured height, weight, and fasting plasma glucose and HbA1c were assessed at a baseline clinic visit (August 2001–April 2004) and yearly thereafter, with the fourth visit occurring between August 2005 and April 2008. All participants signed a consent form approved by their center’s Institutional Review Board. The beneficial effects of the Look AHEAD intervention on diabetes remission and amelioration of other co-morbidities has been previously reported ^9-12^.

Traditional Laboratory Measures

Non-esterified fatty acids (NEFA), total ketones, 3-hydroxybutyric acid (3-HB), and lactate were measured using a Beckman DxC 600 clinical analyzer by standard enzymatic chemistries. Reagents were from Wako Diagnostics (Mountain View, CA), except for lactate, which were from Beckman (Brea, CA). We used QC controls from the corresponding vendors to ensure values were within established ranges. Inter-assay coefficients of variation (CV) were generally <5%. High-sensitivity C-reactive protein (hs-CRP) was measured with Beckman reagents. Glucose and insulin were measured by the same central laboratory for LABS and Look AHEAD. Insulin was measured by a two site immuno-enzymometric assay on a dedicated autoanalyzer (TOSOH 2000). The assay has a sensitivity of 0.5 uU/mL and is linear up to 330 uU/mL. The inter-assay CVs, as determined on quality control samples with low, medium, and high insulin levels, are 2.8%, 2.5% and 2.0%, respectively. Measurement of the relative proportion of glycated hemoglobin and calculation of the HbA1c levels were performed by a dedicated analyzer (TOSOH, Biosciences, Inc., South San Francisco, CA) using non-porous ion exchange high performance chromatography to achieve rapid and precise separation of stable HbA1c from other hemoglobin fractions. The laboratory participates in the National Glycohemoglobin Standardization Program and the method is yearly certified at the level of Laboratory 1 Certification ^13^. The inter-assay CVs for the low and high QC samples are 0.9% and 0.6%, respectively. One study subject on insulin at baseline with missing HbA1c was replaced with the median HbA1c of all other subjects on insulin at baseline.

Metabolomic profiling

A total of 85 metabolites were quantitatively measured in frozen, fasting plasma from baseline (pre-intervention) samples using mass-spectrometry based methods: trimethylamine-N-oxide (TMAO), choline, betaine, amino adipic acid, β-Amino isobutyric acid, branched chain keto acids, 13 bile acids, 15 amino acids, 45 acylcarnitines, 21 ceramides and 29 sphingomyelins.

TMAO, choline and betaine were analyzed by LC-MS/MS as described previously ^14^. Briefly, 50 μl of plasma containing isotopically labeled internal standards TMAO-d9, choline-d9, and betaine-d11 (Cambridge Isotope Laboratories) were precipitated with 400 μl of methanol. After centrifugation, 25 μl of the supernatant were removed and diluted with 100 μl of acetonitrile:methanol (75:25, v/v). The samples were injected onto a Waters Acquity UPLC system coupled to a Waters Xevo TQ-S triple quadrupole mass spectrometer. The analytical column (Waters Acquity UPLC BEH Amide Column, 1.7 μm, 2.1 × 100 mm) was used at 30°C, 10 μl of the sample were injected onto the column, and eluted at a flow rate of 0.3 ml/min. The gradient began with 15% eluent A (10 mM ammonium formate in water, pH=3.5) and was then programmed as follows: 0 to 0.5 min – hold at 15% A, 0.5 to 2.5 min gradient to 40% eluent B (acetonitrile); 2.5 to 3.5 min - hold at 40% B, return to 15% A, and re-equilibrate the column at initial conditions for 1 minute. Mass transitions of *m/z* 76 → 58 (TMAO), 85 → 66 (TMAO-d9), 104 → 60 (choline), 113 → 69 (choline-d9), 118 → 58 (betaine), and 129 → 69 (betaine-d11) were monitored in a positive ion electrospray ionization mode ^14^.

2-Amino adipic acid (2-AAA) and β -amino isobutyric acid (BAIBA) were analyzed by LC-MS/MS. 50 μl of plasma containing isotopically labeled internal standards d3-2-AAA (CDN Isotopes) and d3-BAIBA (Medical Isotopes) were precipitated with 400 μl of methanol. The methanol supernatants were dried and esterified with acidified butanol for 15 min. at 65oC. The samples were reconstituted in 5% methanol and injected onto a Waters Acquity UPLC system coupled to a Waters Xevo TQ-S triple quadrupole mass spectrometer. The analytical column (Waters Acquity UPLC HSS T3 Column, 1.8 μm, 2.1 × 100 mm) was used at 30°C, 10 μl of the sample were injected onto the column, and eluted at a flow rate of 0.4 ml/min. The gradient began with 95% eluent A (0.1 % formic acid in water) and was then programmed as follows: 0 to 4 min – gradient to 20% eluent B (acetonitrile); 4 to 6 min gradient to 90% B; 6 to 7 min - hold at 90% B, return to 95% A, and re-equilibrate the column at initial conditions for 1 minute. Mass transitions of *m/z* 274→ 98 (2-AAA), 277 → 101 (d3-2-AAA), 160 → 86 (β -AIBA), and 163 → 89 (d3-β-AIBA), were monitored in a positive ion electrospray ionization mode.

Branched chain ketoacids (BCKA) were measured by LC-MS/MS as previously described ^15, 16^. Briefly, 30 μl of plasma containing isotopically labeled internal standards KIC-d3, KIV-5C13 (Cambridge Isotope Laboratories), and KMV-d8 (Toronto Research Chemicals) were precipitated with 150 μl of 3M PCA, and BCKA were extracted with ethyl acetate as previously described ^17^. Samples were analyzed on a Waters Xevo TQ-S triple quadrupole mass spectrometer coupled to a Waters Acquity UPLC system.

Amino acids and acylcarnitines were analyzed by flow injection electrospray ionization tandem mass spectrometry (MS/MS) and quantified by isotope or pseudo-isotope dilution using methods described previously ^18, 19^. Briefly, 100 μl of plasma were spiked with a cocktail of heavy-isotope internal standards (Cambridge Isotope Laboratories, MA, USA; CDN Isotopes, Canada) and deproteinated with methanol. Mass spectra for acylcarnitine and amino acid esters were obtained using precursor ion and neutral loss scanning methods, respectively. The data were acquired using a Waters TQ (triple quadrupole) detector equipped with Acquity^TM^ UPLC system and a data system controlled by MassLynx 4.1 operating system (Waters, Milford, MA).

Ceramides and sphingomyelins were analyzed as described previously ^20^. Briefly, plasma samples were spiked with ceramide d18:1/17:0 and sphingomyelin d18:1/12:0 (Avanti, Alabaster, Alabama, USA) and extracted overnight with methanol/chloroform (2:1, v/v) at 50°C. The samples were centrifuged (10 min at 3000g) and supernatants were subjected to alkaline hydrolysis to remove glycero-phospholipids. After neutralization with glacial acetic acid, ceramides and sphingomyelins were extracted by adding chloroform/water (1:1, v/v). The samples were vortexed and centrifuged (10 min at 3000g). The lower layer was transferred to a vial, dried down with N2, and re-suspended in methanol/chloroform (2:1, v/v) containing 5 mM ammonium acetate. Ceramides and sphingomyelins were analyzed by flow injection tandem mass spectrometry for precursors of *m*/*z* 264 and 184 respectively using a Xevo TQS spectrometer (Waters, Milford, MA, USA) and 80% methanol/30 mM ammonium hydroxide as the mobile phase.

We used QC controls with corresponding internal standards for each compound measured. Inter-assay coefficients of variation (CV) were generally <15% for all metabolomics analyses.

Bile acids were measured from human serum samples using ultra performance Liquid Chromatography-tandem Mass Spectrometry (LC-MS/MS) as described ^21^. Bile acids were extracted by spiking 100ul of serum samples with deuterated internal standards and mixing with ten volumes of chilled acetonitrile. LC-MS analysis was performed using an API 4000 triple quadrupole mass spectrometer equipped with an electrospray ionization source operated in the negative ion mode and integrated with an Eksigent UltraLC system (ABSCIEX, Foster City, CA). Quantitative standard curves were used, and deuterated internal standards were used to measure recovery. The lower limits of quantitation for the bile acids ranged from 0.5nM to 10nM. Various levels of quality controls were include: intra-assay precision for the measured bile acids, which ranged from 2.9%-5.3%; intra-assay accuracy which range from 95.4% to 105.9%. The inter-assay precision CV ranged from 3.3% to 4.98%.

Calculation of HOMA-IR and HOMA-B

HOMA-IR (homeostatic model assessment of insulin resistance) was calculated using the following formula: HOMA-IR = $\frac{\text{glucose (mg/dL) }\times\text{insulin (uU/mL)}}{405}$ . HOMA-B (homeostatic model assessment of β-cell function) was calculated as follows: HOMA-B = $\frac{360\times\text{insulin (uU/mL)}}{\text{glucose (mg/dL) }-63}$ %.^22^

HOMA-IR and HOMA-B were not calculated and considered to be ‘missing’ for subjects on insulin therapy at a given timepoint (n=28 at baseline; n=9 at the two years follow-up visit) as it could not be ascertained whether insulin therapy was stopped prior to blood sampling; or with glucose < 63 mg/dL (n=1 at baseline; n=1 at the two years follow-up visit). HOMA-B was calculated only for individuals who were not taking insulin.

**Supplementary Figure: Consort diagram for sustained diabetes remission phenotype sample selection
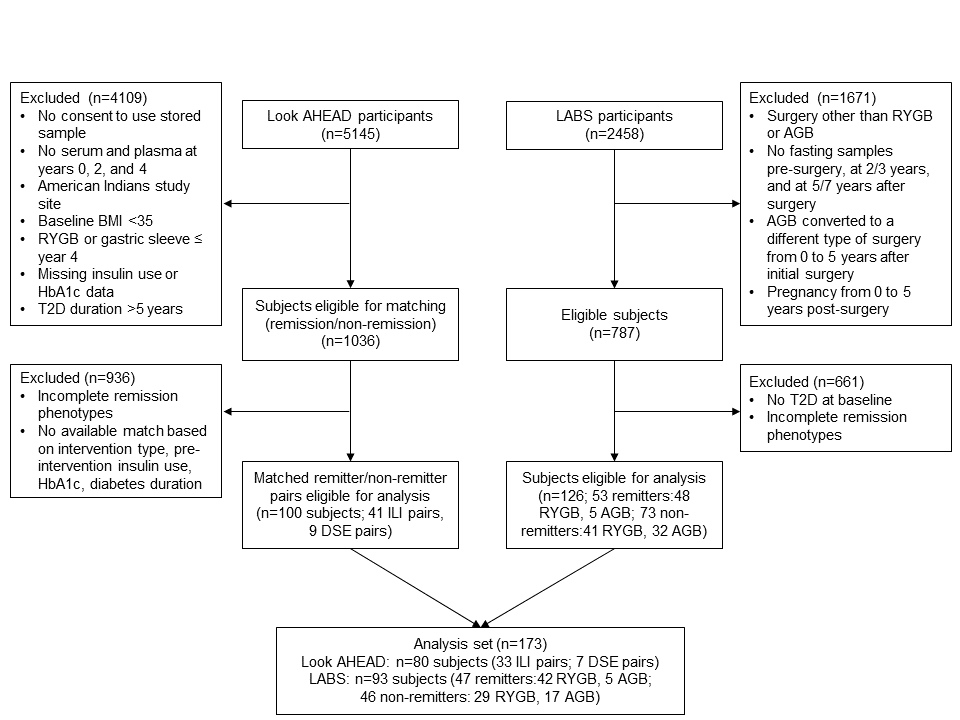
**

**Supplementary Table 1.** Principal components analysis-derived factors, and the primary metabolites in each (|loading|>0.4).

| Factor | Annotation | Primary metabolites |
| --- | --- | --- |
| Factor 1 | Acylcarnitines | AC(C10), AC(C14:1), AC(C12),AC(C8), AC(C16:1), AC(C14:2), AC(C14), AC(C5-DC), AC(C12:1), AC(C10:1), AC(C5), AC(C16), AC(C18:1), AC(C10-OH/C8-DC), AC(C14:1-OH/C12:1-DC), AC(C8:1-OH/C6:1-DC), AC(C18), AC(C6-DC/C8-OH), AC(C18:2), AC(C8:1-DC), AC(C2), AC(C4-DC/Ci4-DC) |
| Factor 2 | Branched-chain and other amino acids | phenylalanine, tyrosine, methionine, valine, leucine/isoleucine, arginine, histidine |
| Factor 3 | Ketone metabolites, acylcarnitines, FFA/NEFA | 3-OH butyrate, total ketones, AC(C4-OH), AC(C2), free fatty acid/nonesterified fatty acid (FFA/NEFA) |
| Factor 4 | Bile acids | taurocholic acid, taurochenodeoxycholic acid, glycocholic acid, taurolithocholic acid, glycochenodeoxycholic acid, taurodeoxycholic acid |
| Factor 5 | Acylcarnitines | AC(C20-OH/C18-DC), AC(C18:1-DC), AC(C18-OH/C16-DC), AC(C16-OH/C14-DC), AC(C18:1-OH/C16:1-DC), AC(C20), AC(C14-OH/C12-DC) |
| Factor 6 | Amino acids | serine, glycine |
| Factor 7 | Bile acids | deoxycholic acid, lithocholic acid, hyodeoxycholic acid |
| Factor 8 | Acylcarnitines | AC(C10:3), AC(C8:1), AC(C10:2),AC(C10:1) |
| Factor 9 | Bile acids | chenodeoxycholic acid, cholic acid, ursodeoxycholic acid |
| Factor 10 | Acylcarnitines | AC(C20:4), AC(C22) |
| Factor 11 | FFA/NEFA | free fatty acid/nonesterified fatty acid |
| Factor 12 | Amino acids and lactate | glutamate/glutamine, lactate |
| Factor 13 | Amino acids and acylcarnitines | ornithine, arginine, AC(C18:1), AC(C18:2) |
| Factor 14 | Betaine, choline | betaine, choline |
| Factor 15 | Amino acids | citrulline, arginine |
| Factor 16 | Bile acids | glycodeoxycholic acid, taurodeoxycholic acid |
| Factor 17 | TMAO, acylcarnitines | TMAO, AC(C12-OH/C10-DC) |
| Factor 18 | Amino acids and acylcarnitines | asparate/asparagine, AC(C5:1) |
| Factor 19 | Amino acids | proline, alanine |
| Factor 20 | Branched-chain ketoacids, amino acids, 2-AAA | KIC, KMV, KIV, valine, leucine/isoleucine, 2-aminoadipic acid (2-AAA) |
| Factor 21 | Acylcarnitines | AC(C4/Ci4) |
|  |  |  |
| CS Factor 1 | Sphingomyelins | Sph(C38:2), Sph(C34:2), Sph(C36:2), Sph(C42:3), Sph(C41:2), Sph(C40:2), Sph(C39:2), Sph(C42:2), Sph(C34:1), Sph(C37:2), Sph(C35:1) |
| CS Factor 2 | Ceramides and sphingomyelins | Cer(d18:1/26:1, Cer(d18:1/26:0), Cer(d18:1/25:0), Cer(d18:1/20:0), Cer(d18:1/18:0), Sph(C44:2), Cer(d18:1/22:0), Sph(C44:1), Cer(d18:1/24:1), Cer(d18:1/24:0), Cer(d18:1/23:0), Cer(d18:1/16:0) |
| CS Factor 3 | Sphingomyelins and glucosylceramides | Sph(C35:3), Sph(C45:1),Sph(C43:3), Sph(C45), GlcCer(d18:1/26:0, Sph(C37:2), Sph(C39:2), GlcCer(d18:1/20:0), GlcCer(d18:1/23:0), Sph(C44) |
| CS Factor 4 | Glucosylceramides, ceramides | GlcCer(d18:1/24:0, GlcCer(d18:1/24:1), GlcCer(d18:1/16:0), GlcCer(d18:1/22:0), GlcCer(d18:1/23:0), GlcCer(d18:1/18:0), Cer(d18:1/16:0), Cer(d18:1/24:1), GlcCer(d18:1/20:0), Sph(C34:1), Cer(d18:1/23:0) |
| CS Factor 5 | Sphingomyelins | Sph(C31:1), Sph(C32:1), Sph(C33:1), Sph(C31), Sph(C35:1), Sph(C37:1) |
| CS Factor 6 | Sphingomyelins | Sph(C43:2), Sph(C43:1), Sph(C35:1), Sph(C37:1) |
| CS Factor 7 | Sphingomyelins | Sph(C40:1), Sph(C42:1), Sph(C41:1), Sph(C38:1), Cer(d18:1/24:0, Sph(C40:2), Sph(C43:1), Cer(d18:1/22:0), Sph(C44:1) |
| CS Factor 8 | Sphingomyelins and ceramides | Cer(d18:1/14:0, Cer(d18:1/23:0), Sph(C44:2) |

Acylcarnitines (AC), ceramides (Cer), glucosylceramides (GlcCer), and sphingomyelins (Sph) are listed by the length of the acyl chain, with the number of any double bonds listed after a colon. DC indicates a dicarboxylic acid; OH, a hydroxide; and i, a branched isomer; the d18:1 designation refers to the particular sphingosine unit.

Branched-chain ketoacids: KIC (Ketoisocaproate), KMV (Ketomethylvalerate), KIV (Ketoisovalerate)

Branched-chain amino acids: valine and leucine/isoleucine.

**Supplementary Table 2. Association of candidate metabolites with diabetes remission, n=173**

|  | **OR (95% CI)** | **p** |
| --- | --- | --- |
| Val | 1 (0.74-1.35) | 0.98 |
| Leu/Ile | 1.04 (0.77-1.41) | 0.78 |
| BCAA sum | 1.02 (0.75-1.38) | 0.91 |
| KIV | 1.11 (0.82-1.51) | 0.49 |
| KIC | 1.19 (0.88-1.62) | 0.27 |
| KMV | 1.14 (0.84-1.55) | 0.4 |
| BCKA sum | 1.17 (0.87-1.6) | 0.31 |
| BCKA/BCAA | 1.19 (0.88-1.62) | 0.27 |
| 2-AAA | 0.96 (0.7-1.3) | 0.79 |

Odds ratios are for 1 standard deviation of the metabolite. An odds ratio (OR) > 1 indicates that higher levels of the tested variable is associated with increased odds of diabetes remission; OR<1 indicates that higher levels are associated with decreased odds of remission.

**Supplementary Table 3. Subgroup analysis of association of clinical features and selected metabolite factors with diabetes remission at 2 and 4/5 years.**

|  | **LABS only** | | | **LookAHEAD only** | | | **RYGB only** | | | **Non-RYGB only** | | |
| --- | --- | --- | --- | --- | --- | --- | --- | --- | --- | --- | --- | --- |
|  | **n** | **OR (95% CI)** | **p** | **n** | **OR (95% CI)** | **p** | **n** | **OR (95% CI)** | **p** | **n** | **OR (95% CI)** | **p** |
| Age (years) | 93 | 0.97 (0.93-1.01) | 0.19 | 80 | 0.99 (0.93-1.06) | 0.75 | 71 | 0.97 (0.92-1.02) | 0.29 | 102 | 1 (0.95-1.05) | 0.9 |
| Ancestry (white vs. all other) | 93 | 1.02 (0.3-3.53) | 0.97 | 80 | 3.27 (1.24-9.24) | 0.019 | 71 | 0.85 (0.16-3.79) | 0.84 | 102 | 2.33 (0.96-6.01) | 0.067 |
| Female | 93 | 0.84 (0.34-2.04) | 0.7 | 80 | 0.57 (0.22-1.44) | 0.24 | 71 | 1 (0.35-2.78) | 0.99 | 102 | 0.54 (0.23-1.23) | 0.14 |
| Total cholesterol * | 93 | 1.39 (0.91-2.18) | 0.13 | 80 | 0.71 (0.44-1.11) | 0.14 | 71 | 1.51 (0.92-2.62) | 0.12 | 102 | 0.84 (0.56-1.25) | 0.4 |
| HDL * | 93 | 0.93 (0.61-1.4) | 0.73 | 80 | 0.78 (0.49-1.21) | 0.27 | 71 | 1.07 (0.66-1.79) | 0.78 | 102 | 0.75 (0.49-1.11) | 0.16 |
| LDL * | 88 | 1.19 (0.78-1.83) | 0.43 | 71 | 0.7 (0.43-1.13) | 0.16 | 66 | 1.24 (0.76-2.08) | 0.4 | 93 | 0.91 (0.6-1.37) | 0.65 |
| Triglycerides * | 93 | 1.52 (0.99-2.46) | 0.069 | 80 | 1.15 (0.73-1.91) | 0.55 | 71 | 1.6 (0.96-2.94) | 0.094 | 102 | 1.15 (0.77-1.77) | 0.5 |
| HbA1c (%) | 93 | 0.26 (0.13-0.45) | 0.000021 | 80 | 0.97 (0.5-1.87) | 0.92 | 71 | 0.26 (0.12-0.5) | 0.00031 | 102 | 0.62 (0.36-1) | 0.064 |
| Glucose * | 93 | 0.38 (0.18-0.69) | 0.0049 | 80 | 1.11 (0.71-1.76) | 0.65 | 71 | 0.33 (0.13-0.66) | 0.0074 | 102 | 0.98 (0.66-1.46) | 0.94 |
| Insulin * | 93 | 0.94 (0.61-1.43) | 0.78 | 80 | 0.99 (0.64-1.55) | 0.98 | 71 | 0.83 (0.49-1.34) | 0.44 | 102 | 1 (0.67-1.48) | 0.98 |
| HOMA-IR | 65 | 1 (0.94-1.08) | 0.98 | 80 | 1 (0.89-1.12) | 0.98 | 51 | 0.99 (0.93-1.07) | 0.76 | 94 | 1 (0.9-1.12) | 0.94 |
| HOMA-B | 65 | 1.01 (1-1.02) | 0.009 | 80 | 1 (0.99-1.01) | 0.89 | 51 | 1.01 (1-1.02) | 0.025 | 94 | 1 (1-1.01) | 0.59 |
| Weight at baseline (kg) | 93 | 1.01 (1-1.01) | 0.09 | 80 | 1 (0.99-1.01) | 0.81 | 71 | 1 (1-1.01) | 0.36 | 102 | 1 (0.99-1.01) | 0.69 |
| % Weight change at 2 years | 93 | 0.93 (0.88-0.97) | 0.00091 | 80 | 0.85 (0.78-0.92) | 0.00011 | 71 | 0.94 (0.88-0.99) | 0.025 | 102 | 0.92 (0.88-0.96) | 0.00055 |
| Taking metformin at baseline | 92 | 2.18 (0.9-5.5) | 0.09 | 80 | 0.18 (0.07-0.47) | 0.00051 | 70 | 2.77 (1-7.94) | 0.052 | 102 | 0.23 (0.1-0.53) | 0.00065 |
| # Non-insulin T2D meds at baseline | 92 | 0.83 (0.52-1.33) | 0.45 | 80 | 0.32 (0.15-0.62) | 0.0014 | 70 | 0.78 (0.43-1.37) | 0.39 | 102 | 0.45 (0.26-0.75) | 0.0035 |
| Factor 2 (BCAA/aromatic AA) | 93 | 1.45 (0.96-2.27) | 0.086 | 80 | 1.37 (0.88-2.2) | 0.17 | 71 | 1.4 (0.86-2.34) | 0.18 | 102 | 1.58 (1.06-2.43) | 0.03 |
| Factor 14 (betaine/choline) | 93 | 0.97 (0.64-1.47) | 0.89 | 80 | 0.38 (0.2-0.66) | 0.0014 | 71 | 0.94 (0.58-1.51) | 0.79 | 102 | 0.53 (0.32-0.81) | 0.0064 |
| CS Factor 4 (glucosylceramides, ceramides) | 91 | 1.58 (1.03-2.54) | 0.045 | 34 | 0.78 (0.37-1.55) | 0.48 | 69 | 1.92 (1.12-3.59) | 0.026 | 56 | 0.9 (0.5-1.54) | 0.7 |

* Odds ratios are for 1 standard deviation of the indicated clinical feature or metabolite.

An odds ratio (OR) > 1 indicates that higher levels of the tested variable is associated with increased odds of diabetes remission; OR<1 indicates that higher levels are associated with decreased odds of remission. Weight loss at 2 years is negative and therefore is associated with an increased odds of remission.

CI: confidence interval. HDL: high-density lipoproteins. LDL: low-density lipoproteins. HbA1c: hemoglobin A1c. HOMA-IR: homeostatic model assessment of insulin resistance. HOMA-B: homeostatic model assessment of β-cell function. RYGB: Roux-en-Y gastric bypass. T2D: type 2 diabetes. BCAA: branched-chain amino acids. AA: amino acids.

**Supplementary Table 4. Association of candidate metabolites with extremes of weight loss, n=151**

|  | **OR (95% CI)** | **p** |
| --- | --- | --- |
| Val | 0.98 (0.71-1.35) | 0.89 |
| Leu/Ile | 0.88 (0.63-1.22) | 0.45 |
| BCAA sum | 0.94 (0.68-1.3) | 0.71 |
| KIV | 1.12 (0.81-1.56) | 0.48 |
| KIC | 0.93 (0.67-1.29) | 0.67 |
| KMV | 0.83 (0.6-1.15) | 0.27 |
| BCKA sum | 0.92 (0.66-1.26) | 0.6 |
| BCKA/BCAA | 0.94 (0.68-1.3) | 0.7 |
| 2-AAA | 0.88 (0.62-1.22) | 0.45 |

Odds ratios are for 1 standard deviation of the metabolite. An odds ratio (OR) > 1 indicates that higher levels of the tested variable is associated with increased odds of weight loss; OR<1 indicates that higher levels are associated with decreased odds of weight loss.

**References**

1. Belle S, Consortium L. The NIDDK Bariatric Surgery clinical Research Consortium (LABS). *Surg Obes Relat Dis* 2005; **1**(2)**:** 145-7.

2. Purnell JQ, Selzer F, Wahed AS, Pender J, Pories W, Pomp A *et al.* Type 2 Diabetes Remission Rates After Laparoscopic Gastric Bypass and Gastric Banding: Results of the Longitudinal Assessment of Bariatric Surgery Study. *Diabetes Care* 2016; **39**(7)**:** 1101-1107.

3. Friedman AN, Wahed AS, Wang J, Courcoulas AP, Dakin G, Hinojosa MW *et al.* Effect of Bariatric Surgery on CKD Risk. *Journal of the American Society of Nephrology* 2018; **29**(4)**:** 1289.

4. Courcoulas AP, King WC, Belle SH, Berk P, Flum DR, Garcia L *et al.* Seven-Year Weight Trajectories and Health Outcomes in the Longitudinal Assessment of Bariatric Surgery (LABS). *JAMA Surgery* 2018; **153**(5)**:** 427-434.

5. Courcoulas AP, Christian NJ, Belle SH, Berk PD, Flum DR, Garcia L *et al.* Weight change and health outcomes at 3 years after bariatric surgery among individuals with severe obesity. *JAMA* 2013; **310**(22)**:** 2416-25.

6. Kelley DE. Action for Health in Diabetes: the Look AHEAD clinical trial. *Current Diabetes Reports* 2002; **2**(3)**:** 207-9.

7. Wadden TA, West DS, Delahanty L, Jakicic J, Rejeski J, Williamson D *et al.* The Look AHEAD Study: A Description of the Lifestyle Intervention and the Evidence Supporting It. *Obesity (Silver Spring)* 2006; **14**(5)**:** 737-752.

8. Unick JL, Beavers D, Jakicic JM, Kitabchi AE, Knowler WC, Wadden TA *et al.* Effectiveness of Lifestyle Interventions for Individuals With Severe Obesity and Type 2 Diabetes. *Diabetes Care* 2011; **34**(10)**:** 2152.

9. Gregg EW, Chen H, Wagenknecht LE, Clark JM, Delahanty LM, Bantle J *et al.* Association of an intensive lifestyle intervention with remission of type 2 diabetes. *JAMA* 2012; **308**(23)**:** 2489- 2496.

10. Gregg EW, Lin J, Bardenheier B, Chen H, Rejeski WJ, Zhuo X *et al.* Impact of Intensive Lifestyle Intervention on Disability-Free Life Expectancy: The Look AHEAD Study. *Diabetes Care* 2018; **41**(5)**:** 1040.

11. Knowler W, Bahnson J, Bantle J, Bertoni A, Bray G, Chen H *et al.* Effect of a long-term behavioural weight loss intervention on nephropathy in overweight or obese adults with type 2 diabetes: a secondary analysis of the Look AHEAD randomised clinical trial. *The Lancet Diabetes & Endocrinology* 2014; **2**(10)**:** 801-809.

12. Gregg EW, Jakicic J, Blackburn G, Bloomquist P, Bray G, Clark J *et al.* Association of the magnitude of weight loss and changes in physical fitness with long-term cardiovascular disease outcomes in overweight or obese people with type 2 diabetes: a post-hoc analysis of the Look AHEAD randomised clinical trial. *The Lancet Diabetes & Endocrinology* 2016; **4**(11)**:** 913-921.

13. Little RR, Rohlfing CL. HbA1c Standardization: Background, Progress and Current Issues. *Laboratory Medicine* 2009; **40**(6)**:** 368-373.

14. Ocque AJ, Stubbs JR, Nolin TD. Development and validation of a simple UHPLC–MS/MS method for the simultaneous determination of trimethylamine N-oxide, choline, and betaine in human plasma and urine. *Journal of Pharmaceutical and Biomedical Analysis* 2015; **109:** 128-135.

15. Glynn EL, Piner LW, Huffman KM, Slentz CA, Elliot-Penry L, AbouAssi H *et al.* Impact of combined resistance and aerobic exercise training on branched-chain amino acid turnover, glycine metabolism and insulin sensitivity in overweight humans. *Diabetologia* 2015; **58**(10)**:** 2324-35.

16. White PJ, Lapworth AL, An J, Wang L, McGarrah RW, Stevens RD *et al.* Branched-chain amino acid restriction in Zucker-fatty rats improves muscle insulin sensitivity by enhancing efficiency of fatty acid oxidation and acyl-glycine export. *Mol Metab* 2016; **5**(7)**:** 538-551.

17. Olson KC, Chen G, Lynch CJ. Quantification of branched-chain keto acids in tissue by ultra fast liquid chromatography-mass spectrometry. *Analytical biochemistry* 2013; **439**(2)**:** 116-122.

18. An J, Muoio DM, Shiota M, Fujimoto Y, Cline GW, Shulman GI *et al.* Hepatic expression of malonyl-CoA decarboxylase reverses muscle, liver and whole-animal insulin resistance. *Nature Medicine* 2004; **10:** 268.

19. Newgard CB, An J, Bain JR, Muehlbauer MJ, Stevens RD, Lien LF *et al.* A branched-chain amino acid-related metabolic signature that differentiates obese and lean humans and contributes to insulin resistance. *Cell Metab* 2009; **9**(4)**:** 311-26.

20. Merrill AH, Jr., Sullards MC, Allegood JC, Kelly S, Wang E. Sphingolipidomics: high-throughput, structure-specific, and quantitative analysis of sphingolipids by liquid chromatography tandem mass spectrometry. *Methods* 2005; **36**(2)**:** 207-24.

21. Haeusler RA, Camastra S, Nannipieri M, Astiarraga B, Castro-Perez J, Xie D *et al.* Increased Bile Acid Synthesis and Impaired Bile Acid Transport in Human Obesity. *The Journal of clinical endocrinology and metabolism* 2016; **101**(5)**:** 1935-1944.

22. Haffner SM, Miettinen H, Stern MP. The homeostasis model in the San Antonio Heart Study. *Diabetes Care* 1997; **20**(7)**:** 1087-92.
